# Aluminium Chloride instead of Ferric chloride for inducing superior sagittal sinus thrombosis to reduce ferromagnetic artifacts on MRI-imaging in experimental models

**DOI:** 10.1038/s41598-024-61885-8

**Published:** 2024-05-14

**Authors:** Maaike Hachenberger, Tobias Braun, Stefan T. Gerner, Laura Suenner, Anne Schänzer, Martin Juenemann, Clemens Mueller, Astrid Wietelmann, Erwin Stolz, Markus Schoenburg, Marlene Tschernatsch, Tibo Gerriets, Hagen B. Huttner, Mesut Yeniguen

**Affiliations:** 1 Department of Neurology, Justus-Liebig University Giessen, Klinikstrasse 33, 35385 Giessen, Germany; 2 Heart and Brain Research Group, 35385 Giessen, Germany; 3 Department of Neurology, Lahn-Dill-Kliniken Wetzlar, 35578 Wetzlar, Germany; 4 Center for Mind, Brain and Behavior (CMBB), University of Marburg and Justus-Liebig-University Giessen, 35032 Marburg, Germany; 5 Institute of Neuropathology, Justus-Liebig University Giessen, 35385 Giessen, Germany; 6 Department of Radiology, Kerckhoff-Klinik Bad Nauheim, 61231 Bad Nauheim, Germany; 7 Scientific Service Group Magnetic Resonance Imaging, Max Planck Institute for Heart and Lung Research, 61231 Bad Nauheim, Germany; 8 Department of Cardiac Surgery, Kerckhoff-Klinik Bad Nauheim, 61231 Bad Nauheim, Germany; 9 Die Neurologen, Private Practice, Frankfurter Strasse 34, 61231 Bad Nauheim, Germany; 10 NeuroCentrum Wetzlar, Sportparkstrasse 2, 35578 Wetzlar, Germany

**Keywords:** Brain, Magnetic Resonance Imaging, Rats, Sinus thrombosis, Stroke, Cerebrovascular disorders, Stroke

## Abstract

Using ferric chloride (FeCl3) to induce experimental superior sagittal sinus (SSS) thrombosis might interfere with magnetic resonance imaging (MRI)-assisted visualization and evaluation of the thrombus, the brain parenchyma, and the quality of the occlusion. The aim of this study was to investigate whether aluminum chloride (AlCl3)-induced thrombosis of the SSS has comparable properties to those of FeCl3 without causing artifacts in MRI. SSS thrombosis was induced in 14 male Wistar rats by exposure of the SSS and subsequent topical application of a filter paper strip soaked in AlCl3 (n = 7) or FeCl3 (n = 7) over a period of 15 min. The animals with AlCl3-induced SSS thrombosis showed a constant and complete occlusion with in histological analysis large thrombi. Blood flow measurements indicated a significant reduction on the first and seventh postoperative day compared to preoperative measurements. MRI enabled visualization and subsequent evaluation of the thrombus and the surrounding parenchyma. In comparison, FeCl3-induced SSS thrombosis could not be evaluated by MRI due to artifacts caused by the paramagnetic properties and increased susceptibility of FeCl3. The occluded sinus and the surrounding area appeared hypointense. The quality of SSS occlusion by AlCl3 was comparable to that of FeCl3. AlCl3 therefore represents a significant alternative substance in experimental SSS thrombosis ideally suited for studies using MRI.

## Introduction

Cerebral and venous sinus thrombosis^1^ is a rather rare and probably underdiagnosed form of stroke^2,3^ constituting about 1% of all strokes.

The annual incidence in the population is about 7 per 1 million in children and about 3–4 per 1 million in adults, with women being much more frequently affected^4^.

The most common but unspecific symptom is headache, often presenting as the only clinical symptom; in about 10% of cases, it is associated with focal neurological signs, epileptic seizures, or impaired consciousness^5^.

In the past, numerous animal studies on cerebral sinus and venous thrombosis have been performed. Different models were used in which sinus thrombosis was induced in various ways in rabbits, gerbils, cats, pigs and rats^6–9^.

SSS thrombosis in rats was induced by topical application of ferric chloride (FeCl3). Depending on the question under study, the duration of the application varied^10,11^.

Parallel to the development and application of magnetic resonance imaging (MRI) in human medicine, MRI has developed into an important tool in basic research. Advantages of this method are the lack of ionizing radiation, the noninvasive character, the high image resolution, and the arbitrary choice of layer thickness. The image quality has improved considerably in recent years.

Measurements by our research group with a 7T magnetic field and a planar surface coil using FeCl3 showed artifacts due to ferromagnetic effects caused by the substance^12^. Due to the artifacts, assessment of the brain parenchyma was only partly possible, and assessment of the thrombus and recanalization was only partly or not at all possible.

In the search for alternative substances, aluminum chloride (AlCl3) has proven to be a potent initiator in the formation of intravascular thrombi^12^. AlCl3 has lower paramagnetic effects in comparison to FeCl3 and therefore enables adequate MRI evaluation.

So far, there is no animal evidence for the use of AlCl3 in connection with the induction of thrombosis of the superior sagittal sinus (SSS). Wolters et al.^12^ proved the successful use of AlCl3 in the thrombosis of the carotid artery in mouse stroke models. The researchers compared the use of FeCl3 and AlCl3 of the same concentration (40%) with the same duration (15 min) of application. A comparable closure was achieved with both substances. The researchers also showed that the use of FeCl3 at low concentrations, starting at 0.5% for T1-weighted imaging and at 0.1% for T2-weighted imaging, has an effect on the MRI signal and leads to hypointensity in the vicinity of the thrombus. Using AlCl3, artifacts were omitted, enabling the differentiation of the thrombus from the surrounding tissue.

This study investigates the use of AlCl3 as an alternative to FeCl3 in an established nonlethal sinus thrombosis animal model. MRI was used to analyze the thrombus, surrounding tissue, and blood flow in the SSS.

### Objectives

In previous studies concerning cerebral and venous sinus thrombosis^1^ in rats, FeCl_3_ was used to induce a thrombus in the SSS. In recent years, the quality of MRI for evaluation improved. Although the resolution of the images increased, artifacts were uncovered while using FeCl_3_, which made it impossible to visualize the thrombus material and the surrounding brain parenchyma. This study establishes an alternative substance (AlCl_3_) that induces an occlusion comparable to that induced by FeCl_3_ without causing MRI artifacts due to paramagnetic properties and increased susceptibility. By visualization of the sinus area and the brain parenchyma, it should be possible to draw a conclusion on the quality of the thrombus, recanalization rates, and parenchyma changes.

## Results

### Functional assessment

#### Neuroscore test

**Figure 1:**
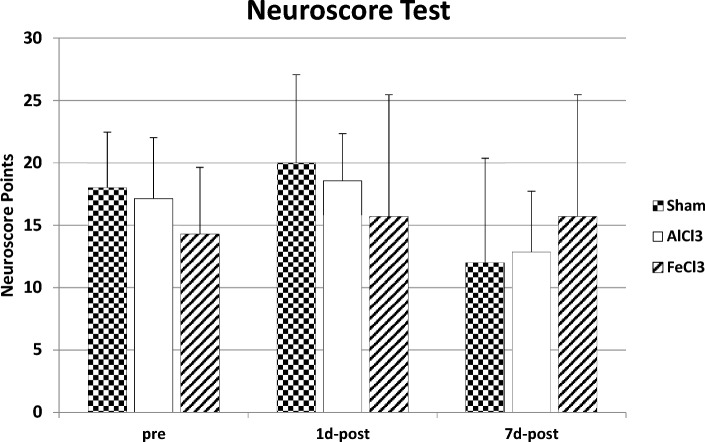
Mean value with standard deviation of the Neuroscore test in each group on all three time of measurements. Higher scores indicate higher overall neurological impairment. There were no significant differences on any time (*p* > 0.05).

Clinical evaluation using the Neuroscore did not show any significant difference on any time of measurement between the three groups (p > 0.05, Kruskal–Wallis-Test). The mean values with standard deviations in the Sham-Group are: pre-measurement 18 ± 4.47, 1day post measurement 20 ± 7.07, 7 day post measurement 12 ± 8.37. AlCl3-group: pre-measurement 17.14 ± 4.88, 1 day post measurement 18.57 ± 3.78, 7day post measurement 12,86 ± 4,88. FeCl3-group: pre-measurement 14,29 ± 5.35, 1day post measurement 15.71 ± 9.76, 7day post measurement 15.71 ± 9.76 (Fig. 1).

#### Rotarod test

**Figure 2:**
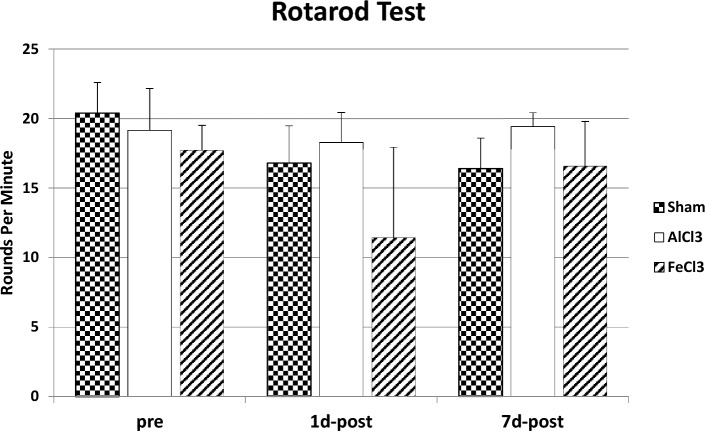
Mean value with standard deviation in rounds per minute of the Rotarod test in each group on all three time of measurements. Higher scores indicate a better performance in the test. There were no significant differences on any time (*p* > 0.05).

The Rotarod Test did not show any significant difference on any time of measurement between the three groups (p > 0.05, Kruskal–Wallis-Test). The mean values with standard deviations of the Sham-group are: pre-measurement 20.4 rpm ± 2.19 rpm, 1day post measurement 16.8 rpm ± 2.68 rpm, 7 day post measurement 16.4 rpm ± 2.19 rpm. AlCl3-group: pre-measurement 19.14 rpm ± 3.02 rpm, 1 day post measurement 18.29 rpm ± 2.14 rpm, 7 day post measurement 19.43 rpm ± 0.98 rpm. FeCl3-group: pre-measurement 17.71 rpm ± 1.8 rpm, 1 day post measurement 11.43 rpm ± 6.5 rpm, 7 day post measurement 16.57 rpm ± 3.21 rpm (Fig. 2).

### Visualization of the thrombus material in the SSS and of the surrounding parenchyma

**Figure 3:**
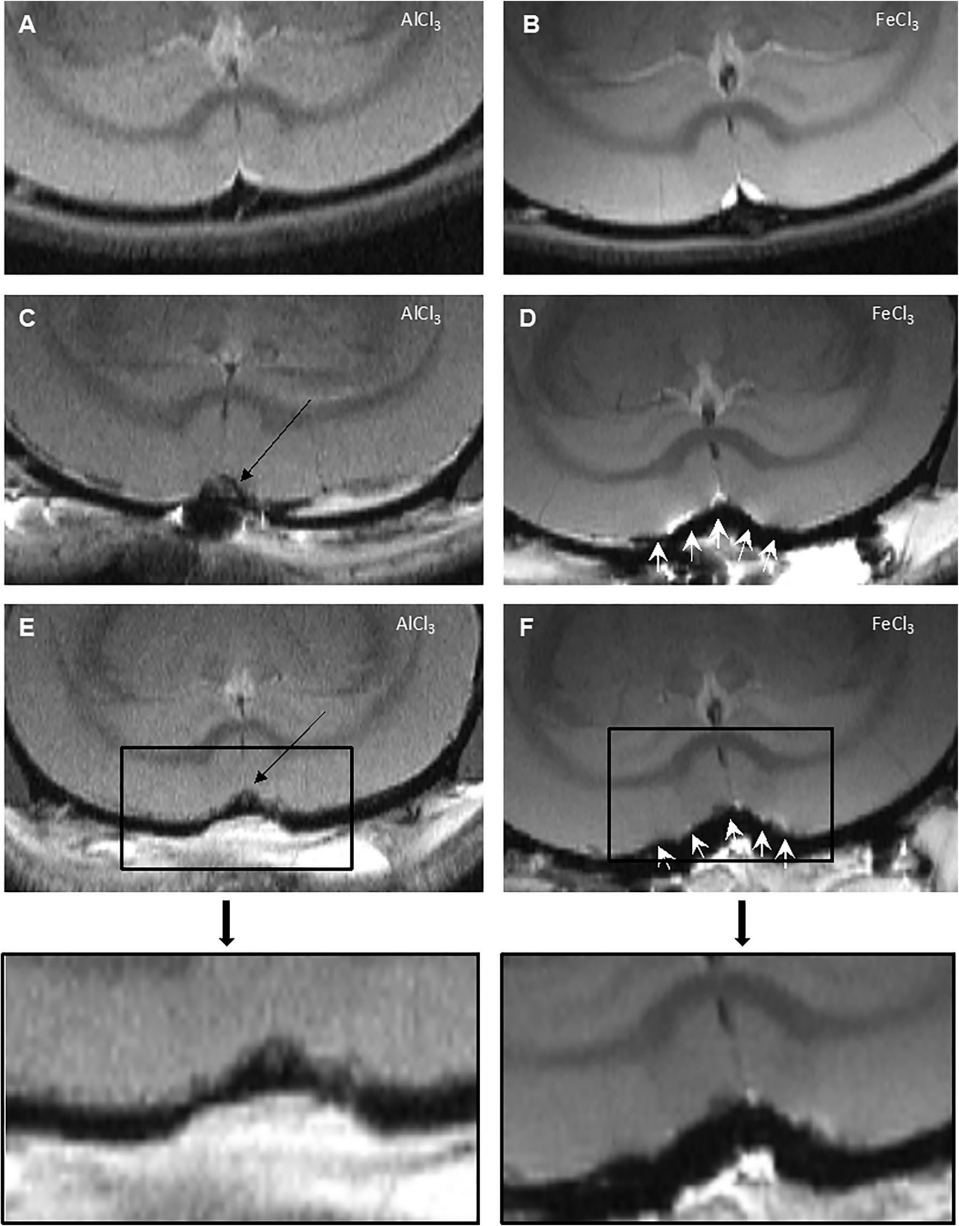
MR Imaging of the Thrombus and the Surrounding Parenchyma. One day prior to surgery, baseline images were taken (**A**, **B**). Using AlCl3, a thrombus was induced in the SSS, marked by the black arrows (**C**, **E**). Thrombus material can be displayed in the proton density images with short TE (18 ms) out of the CPMG sequence on the first (**C**) and on the seventh (**E**) postoperative day. Using FeCl3, disturbance artifacts (white arrows) occurred. The artifacts were due to the residue of FeCl3 in the SSS and concerned the thrombus visualization as well as the representation of the parenchyma.

All animals for which AlCl3 was used showed a complete and constant occlusion of the SSS. The T1- and T2-mapping fast spin echo sequences enabled imaging of thrombus material in the SSS and the surrounding brain parenchyma. One day prior to surgery, MRI was performed to generate baseline images (Fig. 3A,B). On the first and seventh postoperative day, evaluation of the thrombus itself and the surrounding brain region was successful (Fig. 3 C and E).

The use of FeCl3 led to artifacts in T1- and T2-mapping spin echo sequences for each TE and TR, which affected visualization of the thrombus itself and the surrounding parenchyma on the first and seventh postoperative days (Fig. 3D and F). A complete and meaningful representation of the areas of interest was not possible; an evaluation could not be performed, and therefore no statement about the quality of the occlusion can be given.

### Measurement of the blood flow

**Figure 4:**
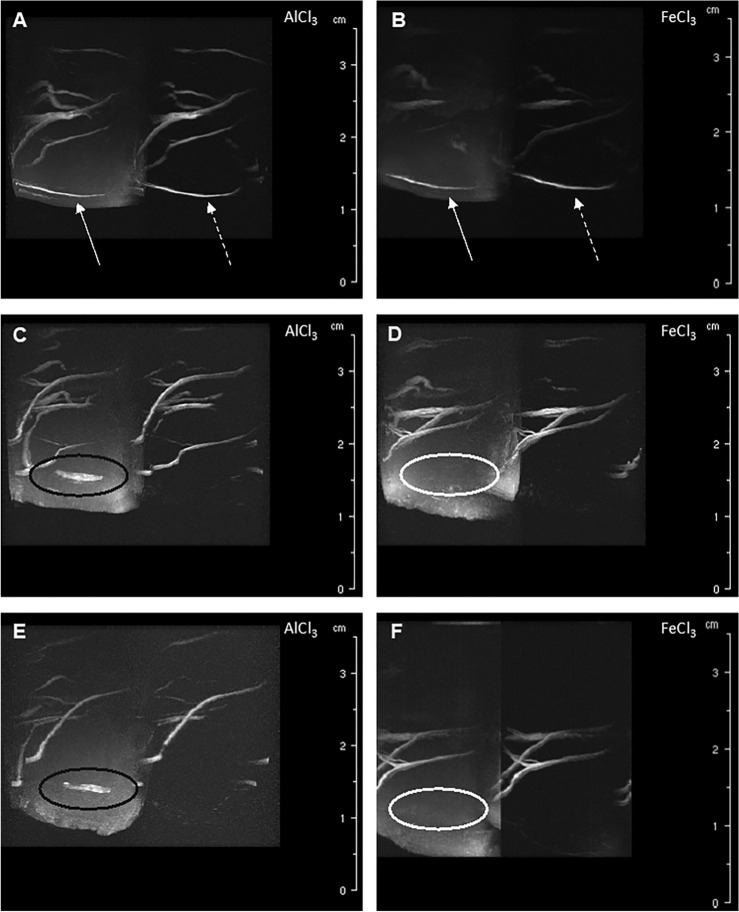
Venous MR Angiography. The first row presents the results of preoperative measurement (**A**, **B**), the middle row the results of measurement on the first postoperative day (**C**, **D**), and the last row the results of measurement on the seventh postoperative day (E, F). Each frame shows a magnitude image (left) and an angiogram (right) (**A**–**F**). (**A**, **B**) In the images of the preoperative measurements, the SSS is shown in the magnitude image (continuous arrow) and in the angiogram (dashed arrow). (**C**, **E**) Representation of thrombus material (black circle) in the magnitude image; the associated angiogram shows no blood flow. (**D**, **F**) Artifacts caused by FeCl3 prevent the representation of the thrombus (white circle) in the magnitude image. Due to the artifacts, the angiogram is not relevant and cannot be used for analysis.

**Figure 5:**
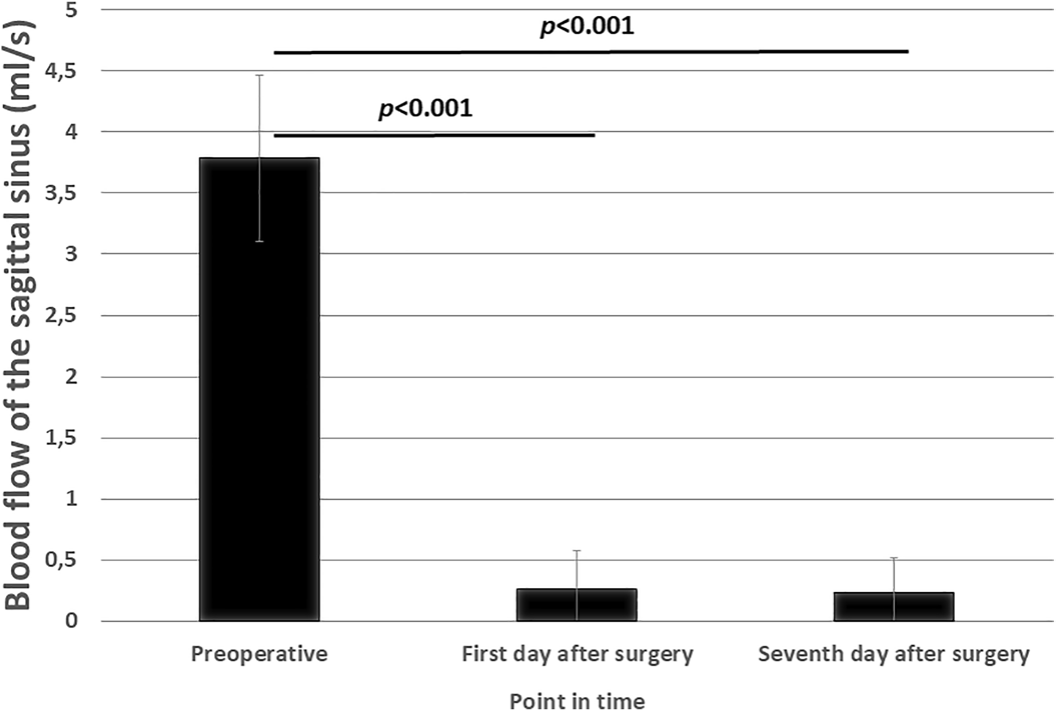
Blood Flow in the Sinus Sagittalis Superior in ml/s. The blood flow was significantly reduced in AlCl3 treated animals at both postoperative times compared to preoperative measurement (*p* < 0.001).

A thrombosis of the SSS leads to complete stagnation of the blood flow. To make a conclusion about the reliability of the use of AlCl3, flow measurements were carried out in the SSS. The use of phase contrast angiography sequences made it possible to visualize the thrombus in the SSS (Fig. 4) and to statistically calculate the flow measurement (Fig. 5).

The blood flow on the first postoperative day was significantly reduced compared to the preoperative measurement, t(6) = 17.06, p =  ≤ 0.001 (Fig. 2C). The blood flow on the seventh postoperative day was significantly reduced compared to the preoperative measurement, t(6) = 15.14, p =  ≤ 0.001 (Fig. 4E).

In the animals that received thrombosis from FeCl3, artifacts appeared in the angiography sequences. The disturbance artifacts affected the signal of both the magnitude image by not showing any thrombus material and of the angiogram at both postoperative times (Fig. 4D and F). An evaluation of the blood flow was not possible.

### Calculation of T1 times in the thrombus

**Figure 6:**
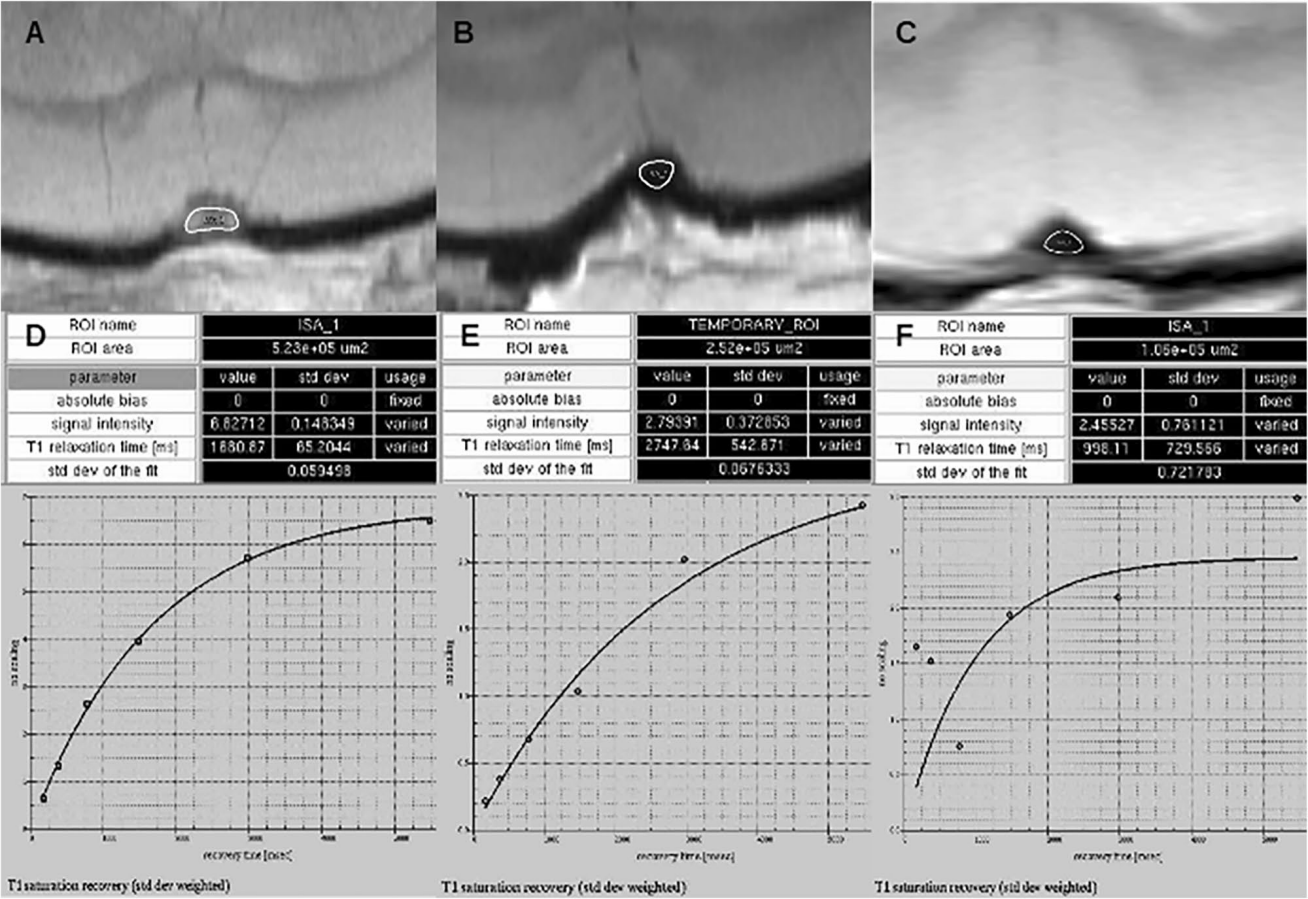
Calculation of T1 Relaxation Times. The occlusion of the SSS by AlCl3 also enables the representation of the thrombus as the calculation of T1 relaxation times with reasonable values (A and D), in D, 1680 ms, for instance. The use of FeCl3 inhibits display of the thrombus or calculating meaningful T1 relaxation times (**B**, **C** and **E**, **F**). Whereas no signal is present in the ROIs of B and C, the least squares evaluation of the measured data in E seems to be of adequate quality, yet the resulting T1 relaxation time of about 2750 ms is far too long for free blood flow and hence far beyond a meaningful threshold for T1 times in the thrombus. In C, no signal in the ROI is detected; correspondingly, the least squares evaluation shows only randomly scattered data (F).

As early changes in the MRI signal during therapy are expected to deliver particularly interesting information concerning the lyse process, T1- and T2-mapping sequences were tested with regard to applicability under the use of AlCl3. Whereas the T2-mapping sequence with short echo times for evaluation of T2 times in the thrombus was only introduced in the animals with occlusion of the SSS with AlCl3, comparison of the evaluation of T1 times between the application of AlCl3 and FeCl3 was possible (Fig. 6).

Whereas the thrombus with the use of AlCl3 was visible and the T1 time calculation resulted in reasonable values (Fig. 6A and D), the use of FeCl3 allowed neither the representation of the thrombus nor the meaningful calculation of T1 times (Fig. 6B,C,E, and F).

### Histology

**Figure 7:**
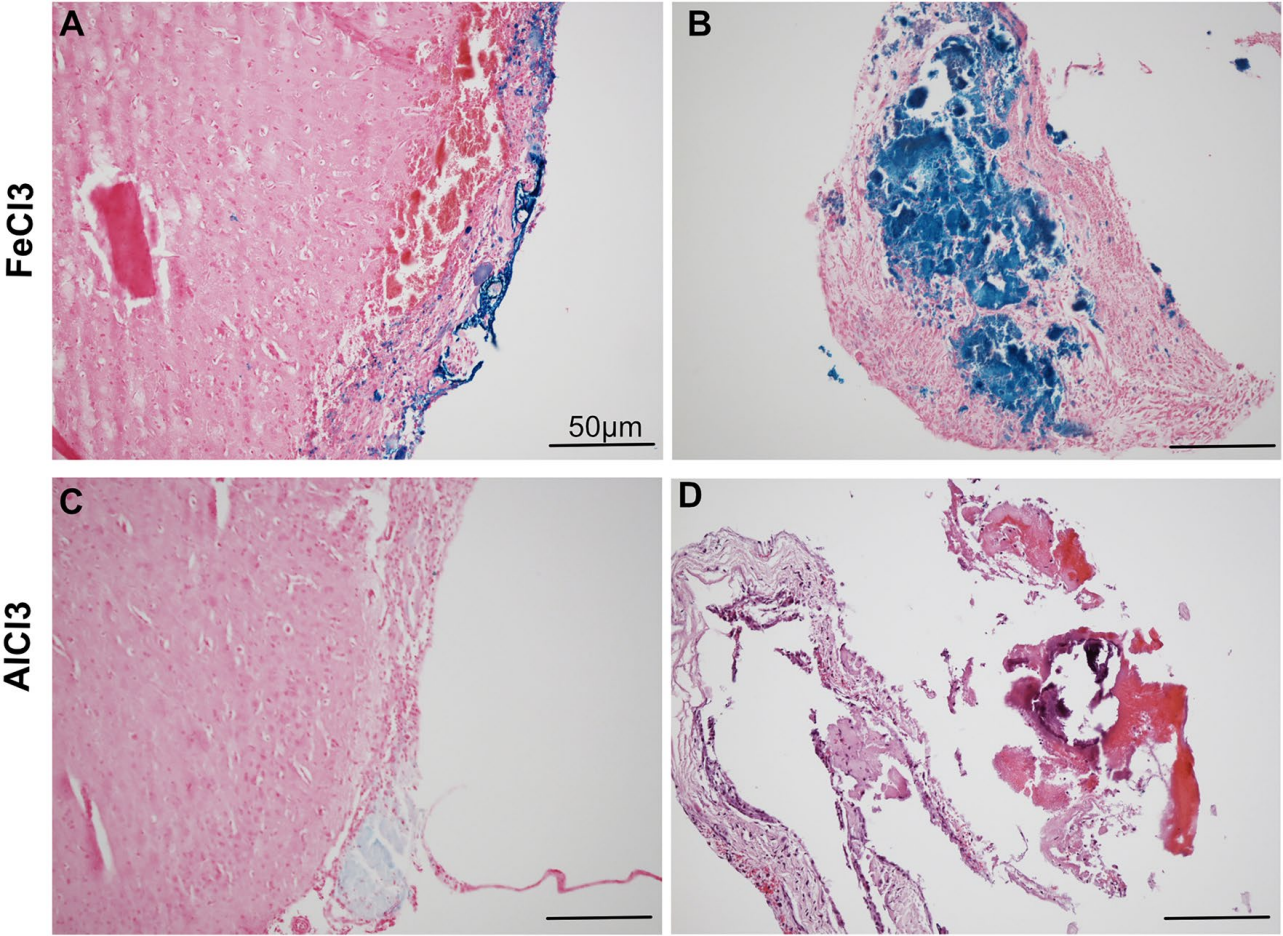
Histology of coronary sections. In FeCl3 treated animals show defects in the cerebral cortex with strong Prussian blue staining (**A**) and large thrombi with strong Prussian blue staining (**B**). In AlCl3 treated animals small cortical defects (**C**) and small thrombi (**D**) are seen without any Prussian blue staining (Magnification × 10).

The results of the histological analysis are shown in detail in Table 1. Animals treated with FeCl3 showed larger defects in the cerebral cortex with strong Prussian blue staining (Fig. 7A). The thrombi were mostly large and stained with Prussian Blue (Fig. 7B). Additional to old blood, fresh blood, macrophages and fibroblasts were seen (Fig. 7B). In comparison the AlCl3 treated animals did show only small cortical defects without any Prussian blue staining. Thrombi were visible only in few sections, containing mainly fresh blood (Fig. 7C/D).

**Table 1:** Histological findings.

Animal No	FeCl_3_	AlCl_3_	Histology	Prussian blue CNS	Prussian blue thrombus
			H&E, EvG		
Edo_S_9 (997,883)	x		macrophages, fresh blood, fibrocytes	+ + +	+ + +
				(And intraventricular)	
Edo_S_1 (986,223)	x		Small thrombus	+ +	+
Edo_S_11 (997,885)	x		macrophages, fresh blood, fibrocytes	+ +	+
Edo_S_13 (1,007,848)	x		macrophages, fresh blood, fibrocytes		
Edo_S_15 (1,007,850)	x		macrophages, fresh blood, fibrocytes	+ +	+ +
Edo_S_16 (1,007,851)	x		macrophages, fresh blood, fibrocytes	+ +	+ +
Edo_S_14 (1,007,849)	x		small thrombus	+	+
Al_8 (10,845,639)		x	no thrombus	–	
Al_10 (1,064,565)		x	no thrombus	( +)	
Al_11 (1,093,690)		x	no thrombus	–	
Al_13 (1,093,692)		x	small thrombus	–	–
Al_14 (1,093,693)		x	no thrombus	–	
Al_15 (1,108,724)		x	no thrombus	–	
Al_17 (1,108,728)		x	small thrombus	–	–

## Discussion

Our aim was to establish an alternative substance to FeCl3 that allows a safe closure of the SSS and simultaneously an evaluation of the results due to missing artifacts and erasures in the MRI.

In this study, thrombosis of the SSS in rats was induced by AlCl3 and FeCl3 and compared using MRI.

AlCl3 induced a constant closure of the SSS by topical application of a soaked filter paper strip. In the T1- and T2-mapping fast spin echo sequences, it was possible to visualize and evaluate the thrombus material, as well as the surrounding brain tissue.

The use of FeCl3 to induce SSS thrombosis led to artifacts in the thrombus area and surrounding tissue in MRI. An evaluation of the thrombus and the brain parenchyma was not possible; a conclusion about recanalization or parenchyma changes in the form of edema or bleeding could not be made.

The artifacts were due to the paramagnetic properties of FeCl3. The protons in the surrounding water molecules in the area of FeCl3 are exposed to an additional local inhomogeneous magnetic field.

This inhomogeneous local field substantially increases the dephasing of transversal magnetization, causing a fast decay of the induced signal. Noise and artifacts are the result. As AlCl3 is also paramagnetic, its magnetic susceptibility is almost 1000 times lower than that of FeCl3 and therefore influences the magnetic field far less.

MR angiography showed the SSS in the sagittal section. Using AlCl3, the occlusion of the SSS could be clearly delimited in the magnitude image.

Using FeCl3, the area around the occluded sinus was displayed as hypointense. The thrombus could not be evaluated in MR angiography due to the paramagnetic effects described above and the increased susceptibility.

Based on the observations described above, AlCl3 should be preferred over FeCl3 for the induction of SSS thrombosis.

To make a conclusion about the quality of the closure and a possible recanalization using AlCl3, a flow measurement was carried out in the SSS.

The flow was significantly reduced on the first and seventh postoperative days compared to the preoperative measurement. The result of the flow measurement on the first and seventh postoperative days was in a very low range, so it can be assumed that there was no flow in the SSS and that there was a constant occlusion of the SSS.

With a constant occlusion, a value of zero is assumed in the flow measurement. The results show a small positive value for the flow on the postoperative days.

The flow measurements were performed by phase contrast methods, evaluated with the inherent software tools of the spectrometer. Even with complete stagnation of the blood flow in the thrombosis, noise results in values different from zero also blood velocity is measured with phase contrast techniques. The values of the measurements on the first and seventh day after surgery aren’t distributed symmetrically around zero flow, as only velocity values in the usual flow direction are used before flow integration as an auxiliary mean to discriminate vessel area from surrounding tissue. Within the standard deviation the flow 1 and 7 days after occlusion is zero.

Using FeCl3, the described paramagnetic effects lead to artifacts that do not enable correct determination of the flow.

Earlier studies by our research group showed that spontaneous recanalizations after SSS thrombosis are to be expected in the first days after occlusion. If there is no early recanalization, a constant occlusion can be expected for weeks^10^.

In the histological evaluation Prussian blue staining shows more distinct cortical changes in the FeCl3 treated animals. Prussian blue has been used to detect iron to visualize thrombi. The FeCl3 deposits are also stained, resulting in a more obvious response to the Prussian blue stain.

In the AlCl3 group, the staining is less pronounced, partly due to the use of the substance and partly due to less damage to the cortex.

Summarized no venous infarcts developed in any of the animals. Venous cortical infarcts develop when thrombosis spreads to bridging veins as a result of consecutive congestion.

The FeCl3 appears to cause parasagittal parenchymal damage and to trigger artifacts on MRI, so that the involvement of bridging veins and congestive infarcts cannot be assessed with certainty.

In our current study, AlCl3 does not appear to trigger artifacts or parenchymal lesions. In future studies, we will focus on the development of cerebral bridging vein thrombosis to further elucidate the pathophysiology of venous infarction.

The present study illustrates limitations to the use of FeCl3 to induce thrombus in the SSS during MRI reprocessing.

Due to artifacts in MRI caused by the limitations of FeCl3, no statements can be made about the thrombus or the surrounding tissue. In regards to comparable thrombus formation properties caused by AlCl3 and FeCl3, our results might also be limited, as we did not histologically analyse thrombus formation in the early period. This was not approved in the proof of concept state of our study by the local research committee to reduce the number of animals. We aim to do this in a future research project.

Using AlCl3, the thrombus in the SSS can be assessed and distinguished from the surrounding tissue; visible artifacts are substantially reduced artifacts. The flow measurement confirms a complete and constant occlusion over the entire observation period. Using our findings, we succesfully applied our model in an experiment comparing edoxaban to enoxaparin for the treatment of SSS thrombosis^13^.

Based on the permanent occlusion of the SSS, the visualization of the thrombus material, and the surrounding brain tissue, the use of AlCl3 to induce SSS thrombosis is a solution in MRI studies.

## Methods

### Animal preparation and experimental design

All animal studies were performed in accordance with institutional guidelines for animal research and were approved by the regional animal care and use committee (Regierungspräsidium Darmstadt, Germany; Az. B10/1001). The study complied with the ARRIVE guidelines.

14 male Wistar rats (Charles River) received analgesia with buprenorphine (Buprenovet, Bayer AG) in a dosage of 0.05 mg/kg body weight subcutaneously (s.c.) half an hour before the anesthetic was administered.

The anesthesia was induced with 5% isoflurane (Isofluran CP, CP-Pharma) in 2 l/min oxygen. The maintenance was performed with 2–2.5% isoflurane in 0.5 l/min oxygen.

The isoflurane concentration was controlled during the entire operation within the specified limits, taking physiological parameters into account.

The body temperature of the animals was kept constant at 37.0 °C throughout the operation using a feedback-heating plate.

SSS thrombosis was induced as previously described^10^.

After the operating area had been shaved and aseptically prepared, the local anesthetic lidocaine (lidocaine-HCl 2% injection solution, B. Braun) was applied s.c. for local anesthesia. A skin incision of about 1.5 cm was made in the median, and the calotte was exposed. The skull bone was drilled down under water cooling so thinly in the midline along the suture that the SSS shone through the bone lamella. The bregma and lambda sutures served as rostral and caudal boundaries. When lifting and removing the bone lamella, care was taken not to damage the dura mater. A filter paper strip soaked with either AlCl3 solution (40%) (n = 7 animals) or FeCl3 solution (40%) (n = 7 animals) was placed on the exposed SSS for a duration of 5 min. Then another strip of filter paper was soaked with the corresponding substance and placed on the exposed sinus for 5 min. This procedure was then repeated a second time, resulting in a 3 times 5 min application. The filter paper strip covered the entire exposed SSS.

To ensure a safe thrombosis of the SSS, a time frame of 15 min was chosen, during which the substance could diffuse through the vessel wall.

Contact of the surrounding brain tissue with the AlCl3 or FeCl3 solution was avoided.

Animals (n = 5) that received a sham operation were used as controls. The filter paper strip was soaked with sodium chloride solution instead of ferric chloride/aluminum chloride.

After removing the last strip of filter paper, the surgical field was carefully rinsed with sterile sodium chloride solution. The skin was closed with a continuous suture. The animals were observed until they completely regained consciousness and then returned to their cages.

The animals were treated with the analgesic buprenorphine at the abovementioned concentration on the day of surgery and the first postoperative day. Furthermore, the animals received metamizol via drinking water from one day prior to surgery up to and including the fifth postoperative day. Seven days after operation, the animals were euthanized and the brains were collected in formalin for histological investigation.

### Functional assessment

To assess the neurological and motor abilities of the animals, a rotarod test and a neuroscore were performed^14^. Both tests were completed preoperatively and on the first and seventh day after surgery.

### MRI imaging

MRI measurements were performed using a 7 Tesla MRI spectrometer (PharmaScan, Bruker) equipped with a 760 mT/m gradient system using a 20 mm 1 H receive-only surface coil together with a 72 mm transmit-only volume resonator. The first measurement was performed preoperatively on a healthy animal. The second measurement took place on the first postoperative day and the third measurement on the seventh postoperative day.

During MRI measurements, the anesthesia of rats was induced using 5% isoflurane at 1 l/min oxygen. Subsequently, the animals were fixed in a cradle with a breathing mask specially built for MRI measurements, and anesthesia was maintained at 1.5–2% isoflurane at 0.5 l/min oxygen. The cradle was placed into the MRI machine until the correct positioning was achieved. The rectal temperature of rats was kept at 37.0 °C using a feedback-controlled water bath. The protocol included T1- and T2-mapping sequences, as well as angiography sequences.

After adjustments of field homogeneity, frequency, and transmit amplitudes, localizer scans in three perpendicular directions were acquired.

For visualization and volumetric analysis of the volume of edema in the surrounding brain parenchyma where appropriate, a T2-CPMG (Carr Purcell Meiboom Gill) mapping sequence was followed: TR = 3800 ms, NEX = 1, matrix = 512 × 256, FOV = 35 × 35 mm^2^, slice thickness = 1 mm, 12 slices, no gap, TE = 18 to 216 ms in steps of 18 ms.

The angiography sequences controlled the degree of occlusion. Due to the low flow velocity in the SSS, the sequences were based on a 3D phase contrast method with an encoding velocity (venc) of 20 cm/s for the detected velocity range: TR = 12 ms, TE = 4,54 ms, NEX = 2; flip angle = 30°, matrix = 256 × 256 × 85, FOV = 30 × 30 × 17 mm^3^, slab thickness = 17 mm. Additionally, a flow map sequence with TR = 15 ms, TE = 4.54 ms, NEX = 8, flip angle = 30°, matrix = 256 × 256 × 11, FOV = 30 × 30 × 11 mm^3^, slab thickness = 11 mm, and venc = 30 cm/s for the flow quantification was included, important particularly in the course of treatment.

Because revascularization represents an advanced status of therapy, a T1- and further T2-mapping sequence were additionally acquired to monitor early changes in the thrombosis. The T1-mapping sequence was based on RARE (Rapid Acquisition with Relaxation Enhancement) with variable repetition times for the reduction of total measurement time compared to inversion or saturation recovery: TE = 9.81 ms, RARE factor = 3, NEX = 1, matrix = 512 × 256, FOV = 35 × 35 mm^2^, slice thickness = 1 mm, 1 slice, TR = 5500, 3000, 1500, 800, 400, and 200 ms. In principle, the use of variable repetition times enables the use of multi-slice acquisition, which was not used in this study because of strong crossover effects between the slices, resulting in strongly altered T1 times compared to single slice acquisition.

For T2-mapping of the thrombosis, a CPMG sequence was also used, yet using a significantly reduced echo spacing due to the manifest lower T2 times in the thrombosis in contrast to brain parenchyma: TR = 2000 ms, NEX = 2, matrix = 256 × 256, FOV = 35 × 35 mm^2^, slice thickness = 1 mm, 12 slices, TE = 4.18 to 104.53 ms in steps of 4.18 ms.

### Histology

After euthanizing the animals, the brains were extracted and fixed with 4% formaline and embedded in paraffin and sections were cut using a formed container to equal every section. The 6µm thick coronal sections were then stained with H&E using a tissue stainer Medite. Elastica van Gieson (EvG) and Prussian blue were stained using a Ventana Bench Mark special stainer. The sections were analysed semi quantitative with a Nikon Eclipse 80i equipped with a digital camera DSFi1.

### Quantitative image analysis

The calculated images of the different sequences were analyzed with a suitable software program (Paravision 6.0.1 Image Display and Processing, Bruker).

Based on the least squares method, T1 and T2 maps were calculated to evaluate possible injury in the surrounding parenchyma and changes of the thrombosis during treatment.

A region of interest for flow measurement was defined in the SSS. Based on the angiography sequences, a sagittal image was created showing vessels in which blood flow took place.

### Data evaluation and statistical analysis

All data in the text and figures are given as mean ± standard error of mean (SEM). Data were tested for normal distribution and variance homogeneity. The Kruskal–Wallis-Test was used to test the functional assessments. A t-test for dependent samples was then performed. The values of the 3 time points (preoperative, 1 day postoperative, 7 days postoperative) were compared, and p < 0.05 was considered statistically significant. The data analysis program SPSS (IBM) was used for evaluation.

### Ethics approval and consent to participate

All animal studies were performed in accordance with institutional guidelines for animal research and were approved by the regional animal care and use committee (Regierungspräsidium Darmstadt, Germany; Az. B10/1001).

## Data Availability

The authors declare that all relevant data is in this manuscript. Individual data can be provided upon reasonable request via the corresponding author.
